# Platelet collagen receptor Glycoprotein VI‐dimer recognizes fibrinogen and fibrin through their D‐domains, contributing to platelet adhesion and activation during thrombus formation

**DOI:** 10.1111/jth.13919

**Published:** 2018-01-15

**Authors:** I. Induruwa, M. Moroi, A. Bonna, J.‐D. Malcor, J.‐M. Howes, E. A. Warburton, R. W. Farndale, S. M. Jung

**Affiliations:** ^1^ Department of Clinical Neurosciences University of Cambridge Cambridge UK; ^2^ Department of Biochemistry University of Cambridge Cambridge UK

**Keywords:** fibrin, fibrin fragment D‐dimer, fibrinogen fragment D, platelet membrane glycoprotein VI, platelet membrane glycoproteins, receptors, collagen

## Abstract

**Essentials**

- Glycoprotein VI (GPVI) binds collagen, starting thrombogenesis, and fibrin, stabilizing thrombi.
- GPVI‐dimers, not monomers, recognize immobilized fibrinogen and fibrin through their D‐domains.
- Collagen, D‐fragment and D‐dimer may share a common or proximate binding site(s) on GPVI‐dimer.
- GPVI‐dimer–fibrin interaction supports spreading, activation and adhesion involving αIIbβ3.

**Summary:**

**Background**

Platelet collagen receptor Glycoprotein VI (GPVI) binds collagen, initiating thrombogenesis, and stabilizes thrombi by binding fibrin.

**Objectives**

To determine if GPVI‐dimer, GPVI‐monomer, or both bind to fibrinogen substrates, and which region common to these substrates contains the interaction site.

**Methods**

Recombinant GPVI monomeric extracellular domain (GPVI_ex_) or dimeric Fc‐fusion protein (GPVI‐Fc_2_) binding to immobilized fibrinogen derivatives was measured by ELISA, including competition assays involving collagenous substrates and fibrinogen derivatives. Flow adhesion was performed with normal or Glanzmann thrombasthenic (GT) platelets over immobilized fibrinogen, with or without anti‐GPVI‐dimer or anti‐αIIbβ3.

**Results**

Under static conditions, GPVI_ex_ did not bind to any fibrinogen substrate. GPVI‐Fc_2_ exhibited specific, saturable binding to both D‐fragment and D‐dimer, which was inhibited by mFab‐F (anti‐GPVI‐dimer), but showed low binding to fibrinogen and fibrin under our conditions. GPVI‐Fc_2_ binding to D‐fragment or D‐dimer was abrogated by collagen type III, Horm collagen or CRP‐XL (crosslinked collagen‐related peptide), suggesting proximity between the D‐domain and collagen binding sites on GPVI‐dimer. Under low shear, adhesion of normal platelets to D‐fragment, D‐dimer, fibrinogen and fibrin was inhibited by mFab‐F (inhibitor of GPVI‐dimer) and abolished by Eptifibatide (inhibitor of αIIbβ3), suggesting that both receptors contribute to thrombus formation on these substrates, but αIIbβ3 makes a greater contribution. Notably, thrombasthenic platelets showed limited adhesion to fibrinogen substrates under flow, which was further reduced by mFab‐F, supporting some independent GPVI‐dimer involvement in this interaction.

**Conclusion**

Only dimeric GPVI interacts with fibrinogen D‐domain, at a site proximate to its collagen binding site, to support platelet adhesion/activation/aggregate formation on immobilized fibrinogen and polymerized fibrin.

## Introduction

Myocardial infarction and ischemic stroke, leading causes of disability and death worldwide 1, usually result from atherosclerotic plaque rupture, leading to arterial thrombus formation followed by distal tissue infarction; platelets are crucial for the development of such thrombi. Disruption of the thrombo‐protective endothelial layer within blood vessels during plaque rupture exposes collagen and tissue factor (TF). Platelet GPIb‐IX‐V binds to von Willebrand factor (VWF) deposited on exposed subendothelial collagens, slowing them down so Glycoprotein VI (GPVI) can bind to the collagen, which initiates a signaling cascade leading to thrombus formation 2, 3. A dimer consisting of two GPVI‐monomers is the functionally active form of this receptor, which is constitutively present (about 20% of the total GPVI) on resting platelets 4. GPVI‐dimers recognize Gly‐Pro‐Hyp (GPO) peptide repeats on collagen 5, 6 and once platelets adhere via GPVI, activation of integrin α2β1 supports firm adhesion to collagen 7.

The subsequent growth and stability of a clot relies on coordinated activation of both the collagen pathway and TF (tissue factor) pathway 8, which can also form thrombi independent of collagen exposure 9, relying on thrombin (factor IIa) generation via the proteolytic extrinsic coagulation cascade. Thrombin generates insoluble fibrin from soluble circulating fibrinogen, which polymerizes and acts to stabilize the growing clot via platelet receptor integrin αIIbβ3 10, 11.

Human fibrinogen molecules are comprised of six disulfide‐linked polypeptide chains (Aα, Bβ, γ)_2_ organized as outer D‐domains containing the C‐termini of the three chains, connected to a central E‐domain. Thrombin cleaves fibrinopeptides A and B from the N‐termini of the α‐ and β‐chains within the E‐domain, exposing new binding sites that allow fibrin polymerization through linking to one or two D‐domains in an end‐to‐middle configuration 12. D‐domain γ‐chains undergo reciprocal crosslinking mediated by FXIIIa, leading to a strengthened fibrin mesh 13. The peptide Gly‐Pro‐Arg‐Pro (GPRP), which binds to the fibrin polymerization sites in the D‐domain, inhibits its binding to an adjacent E‐domain, leading to formation of non‐crosslinked, or monomeric fibrin 14. Plasmin can act on fibrinogen to release D‐ or E‐domains separately, or on polymerized fibrin to release D‐dimer, typically consisting of two FXIIIa crosslinked D‐domains from an original fibrinogen molecule 15.

Initially, αIIbβ3 is converted to its high‐affinity form, enabling binding to fibrinogen and fibrin, leading to platelet aggregation, thrombus growth and formation of a hemostatic plug 16. The complex role that αIIbβ3 plays in thrombus formation has already been extensively investigated, but translation of its inhibition to an antiplatelet agent has not been successful in conditions such as ischemic stroke 17.

It has been long suspected that GPVI plays a role in thrombus growth independent of collagen exposure through ligands such as thrombin 18, 19, laminin 20, fibronectin 21, 22 and vitronectin 23. Recently, it has been reported that GPVI interacts with polymerized fibrin to facilitate thrombus growth 24, 25, 26.

The present study provides evidence indicating that the collagen‐binding, dimeric form of GPVI, is also able to recognize the D‐domain, a region universal to fibrinogen, D‐fragment (resulting from proteolytic cleavage of fibrinogen), D‐dimer, and monomeric and polymerized fibrin. The binding site on GPVI‐dimer for the D‐domain is proximate to its collagen‐binding site and GPVI‐dimer assists αIIbβ3 in thrombus growth and stabilization, suggesting that GPVI‐dimer could be the central platelet receptor linking both collagen and TF pathways in thrombosis.

## Materials and methods

### Fibrinogen/fibrinogen derivatives

Figure S1 summarizes the commercial preparations of human fibrinogen and derivatives used; SDS‐PAGE confirmed the manufacturer's stated purity. Preliminary studies showed that all the listed fibrinogens gave similar results. We used Fibrinogen 3 (Fbg‐3) in this study because it was plasminogen‐, VWF‐ and fibronectin‐ depleted, but not factor XIII– depleted.

### Recombinant GPVI‐dimer and ‐monomer

GPVI_ex_ is a recombinant protein comprising the extracellular domain of GPVI, including the collagen binding portions D1D2 and most of the highly glycosylated extracellular stem portion. GPVI‐Fc_2_ is a fusion protein of the GPVI extracellular domain (same amino acid sequence as GPVI_ex_) and the Fc domain of IgG, which spontaneously dimerizes ([GPVI‐Fc]_2_, abbreviated as GPVI‐Fc_2_). Both proteins were designed and characterized by M. Moroi and colleagues 4, 27.

### Collagen substrates (synthesized and crosslinked in‐house)

CRP‐XL (crosslinked collagen‐related peptide; GCO‐(GPO)_10_‐GCOG‐NH_2_) is a GPVI‐specific agonist 28 and GPP10‐XL is its inactive analogue (GCP‐(GPP)_10_G‐NH_2_) 5.

### GPVI‐dimer‐specific antibodies

mFab‐F is a GPVI‐dimer‐specific inhibitory Fab 3 that recognizes the dimers on resting platelets. GPVI‐dimer‐specific non‐inhibitory 204‐11 Fab 4 recognizes the GPVI‐dimers on resting and activated platelets. Both antibodies were developed by M Moroi and colleagues.

### Immobilization of fibrinogen substrates and collagens

For ELISA, static adhesion, confocal microscopy and flow adhesion, respectively, 96‐well black optical polymer‐bottom ELISA plates (Nunc A/S, Roskilde, Denmark), Maxisorp ELISA plates (Nunc A/S), glass‐bottomed dishes (10‐mm, No. 0; MatTek, Ashland, MA, USA) or glass slides were incubated with fibrinogen or collagenous substrates in phosphate‐buffered saline (PBS; for all substrates except Horm, which was diluted in supplied diluent), overnight at 4 °C; details are provided in the figure legends. The immobilized surfaces were blocked by incubating with 1% bovine serum albumin (BSA)/PBS for 1 h, washed twice with PBS, and used for experiments.

*Polymerized fibrin (pFibrin)* was prepared by adding thrombin (2 U mL^−1^; Sigma‐Aldrich, Dorset, England) to Fbg‐3 (10 μg mL^−1^ unless otherwise stated), and incubated for 30 min at RT, aliquoted to the surface to be coated and left overnight at 4 °C. The coated surface was blocked with 1% BSA, treated with hirudin or protease inhibitor cocktail for 10 min to inhibit active thrombin, and washed with PBS. The presence of fibrin strands was confirmed by staining with anti‐fibrin (UC45)/Alexa‐Fluor‐Plus 555 anti‐mouse IgG (ThermoFisher Scientific, Warrington, UK); Fig. 4(B) shows a representative image.

Immobilized *monomeric fibrin (mFibrin)* was prepared by adding GPRP (2 mm) to Fbg‐3 (10 μg mL^−1^, unless stated otherwise) and incubated for 30 min at RT before adding thrombin as above; other procedures are the same as for pFibrin.

### ELISA analysis of GPVI‐dimer and GPVI‐monomer binding to fibrinogen and its derivatives

GPVI binding to immobilized substrates was measured by ELISA as described previously 4. Bound GPVI was measured by reacting the wells with 1G5 (mouse anti‐pan GPVI Mab; Biocytex, Marseille, France) 20, 29 followed by IRDye^®^800CW goat anti‐mouse IgG (Licor Biosciences, Cambridge, UK) or Alexa‐fluor 647‐conjugated anti‐human Fc (BioLegend, London, UK).

### Washed platelets

ACD‐anticoagulated blood from healthy volunteers or the Glanzmann thrombasthenic (GT) patient was obtained with informed consent, in accordance with the Treaty of Helsinki. Washed platelets were prepared as described previously 30 and resuspended in HEPES‐Tyrode's buffer (HT: 134 mm NaCl, 0.34 mm Na_2_HPO_4_, 2.9 mm KCl, 12 mm NaHCO_3_, 10 mm *N*‐2‐hydroxyethylpiperazine‐*N*’‐2‐ethanesulfonic acid, 5.5 mm glucose, pH 7.3).

### Other platelet assays

Aggregometry was performed with a PAP‐8E aggregometer (Bio/Data Corporation, Alpha Laboratories (UK distributor), Hampshire, UK); details are given in the legend for Fig. 5. Flow cytometry was performed with an Accuri C6 flow cytometer (BD, Oxford, UK) on washed platelets; details are given in the legend for Fig. 3. Static platelet adhesion (Fig. 3) experiments were performed as described previously 4 to determine the effects of mFab‐F and Hip8 (anti‐αIIbβ3) on adhesion of washed platelets to fibrinogen‐substrate‐coated or collagen‐coated ELISA plates.

### Confocal imaging

Washed platelets (3 × 10^7^ mL^−1^ in HT/2 mm MgCl_2_) were allowed to adhere on fibrinogen‐substrate‐coated MatTek dishes for 1 h at 37 °C and formalin fixed as described previously 31. Pre‐ and post‐staining details are provided in the figure legends. Stained samples were imaged under an Olympus FV300 IX81 laser‐scanning confocal microscope (Olympus, Essex, UK), with a 60x oil immersion objective).

### Effect of anti‐GPVI‐dimer antibodies on platelets under flow

Flow adhesion experiments were performed with normal or GT blood and image acquisition was carried out as described 32; details are given in the figure legends. After 5 min, Z‐stacks (ΔZ = 0.69‐μm increments from the coverslip plane) were collected and analyzed by ImageJ1.35 (National Institutes of Health). The coverslip plane was defined as the Z‐plane with the largest fluorescent platelet area and used to calculate the surface area coverage (SA; μm^2^). Thrombus volume (μm^3^) was calculated as the sum of the detected surface areas of all images in the Z‐stack, multiplied by ΔZ. Mean thrombus height (MTH) was calculated as thrombus volume/field area.

### Data analyses

Non‐linear regression (one‐site model) to obtain dissociation constants (*K*_d_) and statistical analyses was performed with Prism version 7.2 (GraphPad Software, San Diego, CA, USA).

## Results

### Dimeric, but not monomeric, GPVI binds to immobilized D‐fragment and D‐dimer with high affinity

**Figure 1:**
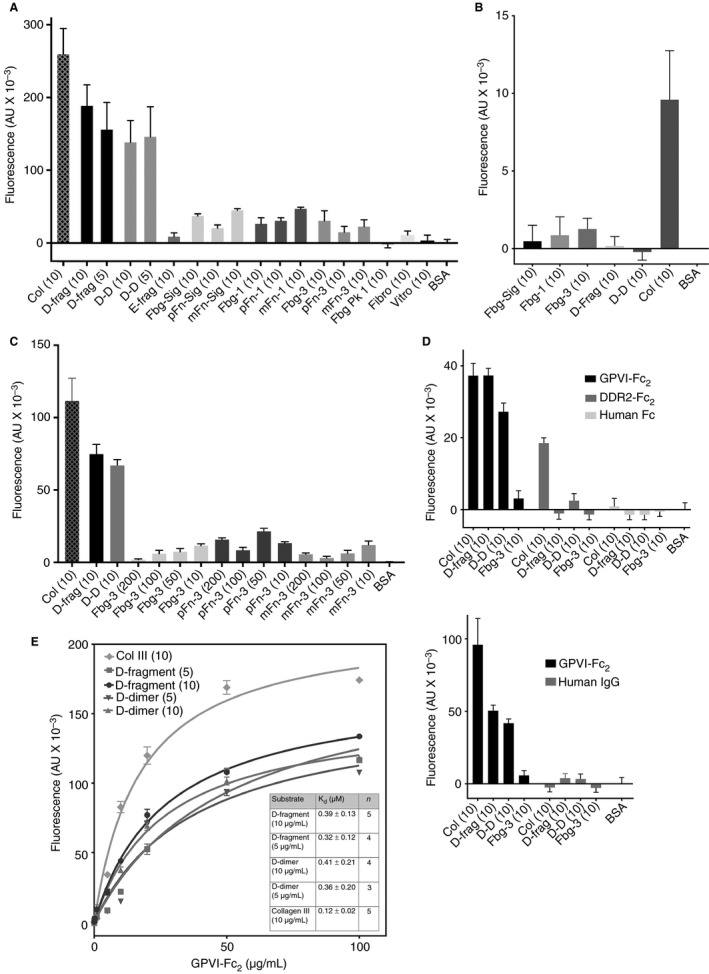
Interaction of GPVI‐Fc_2_ and GPVI_ex_ with immobilized fibrinogen derivatives. The following abbreviations were used in the graphs: collagen type III (Col); D‐fragment (D‐frag); D‐dimer (D‐D); E‐fragment (E‐frag); fibrinogen‐Sigma (Fbg‐Sig), pFibrin‐Sigma (pFn‐Sig), mFibrin‐Sigma (mFn‐Sig); fibrinogen‐1 (Fbg‐1), pFibrin‐1 (pFn‐1), mFibrin‐1 (mFn‐1); fibrinogen‐3 (Fbg‐3), pFibrin‐3 (pFn‐3), mFibrin‐3 (mFn‐3); fibrinogen peak 1 (Fbg Pk1); fibronectin (Fibro); vitronectin (Vitro); BSA (bovine serum albumin). Binding of GPVI‐Fc_2_ (recombinant GPVI‐dimer), GPVI_ex_ (recombinant GPVI monomer), DDR2‐Fc_2_, human Fc and human IgG to immobilized fibrinogen derivatives was measured by ELISA (enzyme‐linked immunosorbent assay) as detailed in the Methods. Collagen type III (Col) was used as a positive control. The numbers in parentheses indicate the concentrations of substrate used to coat the ELISA plate wells in μg mL^−1^ in PBS. Non‐specific binding was determined with BSA alone, which was subtracted from the total binding to obtain specific binding. (A) Fibrinogen‐Sigma, fibrinogen‐1, fibrinogen‐3 and fibrinogen peak 1 are the fibrinogens described in Figure S1 and the corresponding polymerized (pFibrin) and monomeric fibrins (mFibrin) produced from thrombin +/− GPRP addition, respectively. Fluorescence values shown are the mean ± SEM, from five different experiments, with duplicate repeats in each. GPVI‐Fc_2_ (50 μg mL^−1^ shown) shows high levels of binding to D‐fragment, D‐dimer and col III. In contrast, there is only very low binding to fibrinogen, mFibrin and pFibrin and no binding to E‐fragment, fibrinogen peak 1, fibronectin or vitronectin. (B) GPVI_ex_ (50 μg mL^−1^) showed no binding to immobilized fibrinogen‐Sigma, fibrinogen‐1, fibrinogen‐3, D‐fragment or D‐dimer with some binding to collagen III, suggesting that the GPVI monomer is unable to recognize the D‐domain. (C) No increase in binding to GPVI‐Fc_2_ is seen with fibrinogen‐3 (Fbg‐3) or pFibrin‐3 and mFibrin‐3 produced from it, at higher concentrations (Fbg‐3; 10–200 μg mL^−1^), whereas collagen III, D‐fragment and D‐dimer bind at 10 μg mL^−1^. (D) Human Fc shows no binding to the fibrinogen derivatives and no binding to col III. Immobilized DDR2‐Fc_2_ (20 μg/mL) binds collagen as expected, but neither DDR2‐Fc_2_, human Fc (20 μg/mL) nor human IgG (25 μg mL^−1^) bind to fibrinogen, whereas matched concentrations of GPVI‐Fc_2_ bind well to D‐fragment, D‐dimer and collagen. (E) Kinetic analyses of the binding of GPVI‐Fc_2_ to D‐fragment and D‐dimer to determine *K*_d_ (dissociation constant) values. GPVI‐Fc_2_ exhibits saturable binding to both D‐fragment and D‐dimer (coated at two different concentrations), with *K*_d_ values (data fit to a non‐linear regression, one‐site model) shown in the inset. The *K*_d_ values for D‐fragment and D‐dimer are similar and they are comparable to the *K*_d_ value for col III.

We measured GPVI‐Fc_2_ binding to immobilized fibrinogen derivatives and collagen III (col III) (Fig. 1A; mean ± SEM, 3 experiments, duplicate replicates). GPVI‐Fc_2_ showed high levels of binding to D‐fragment and D‐dimer, but did not bind E‐fragment. There was very low but measurable binding to fibrinogen, pFibrin and mFibrin. GPVI‐Fc_2_ binding to D‐fragment or D‐dimer was specific and saturable, their *K*_d_ values being higher but similar in magnitude to that for col III (Fig. 1E). At all coating concentrations (10–200 μg mL^−1^, Fig. 1C), fibrinogen, mFibrin or pFibrin binding was too low and scattered to determine *K*_d_. *B*_max_, which depends on the amount of immobilized substrate, cannot be determined by ELISA.

Monomeric GPVI, GPVI_ex_ (0–50 μg mL^−1^), did not bind D‐fragment, D‐dimer or fibrinogen and exhibited feeble col III binding compared with GPVI‐Fc_2_, as previously reported 4 (Fig. 1B).

Furthermore, mFab‐F significantly reduces GPVI‐Fc_2_ binding to D‐fragment (*P < *0.01), D‐dimer (*P <* 0.01) and collagen type III (*P < *0.05), compared with a non‐specific human Fab (hFab; Jackson ImmunoResearch) control (Fig. 5B). These results suggest that the binding site for GPVI lies in the D‐domain, universally present in fibrinogen, D‐dimer, mFibrin and pFibrin, for which only GPVI‐dimer has good affinity.

Human Fc and DDR2‐Fc_2_ (recombinant Fc‐fusion protein of discoidin domain‐containing receptor 2, another collagen‐binding protein 33) and human IgG did not bind to any of the fibrinogen substrates, whereas DDR2‐Fc_2_ bound to collagen as expected, confirming the specificity of our assay to the GPVI portion of GPVI‐Fc_2_ (Fig. 1D).

### Collagenous substrates inhibit the binding of GPVI‐dimer to D‐fragment and D‐dimer, suggesting proximity of binding sites

**Figure 2:**
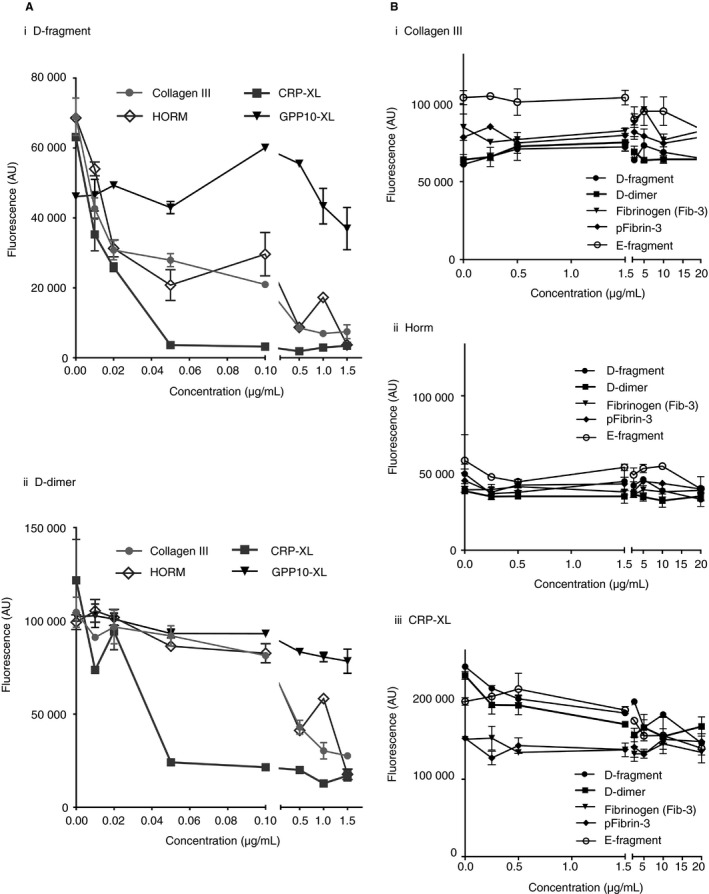
Collagenous substrates compete with GPVI‐dimer binding to immobilized D‐fragment and D‐dimer, but fibrinogen substrates in solution do not displace binding of GPVI‐dimer to immobilized collagen substrates. (A) Collagenous substrates inhibit the binding of GPVI‐Fc_2_ to D‐fragment and D‐dimer. D‐fragment or D‐dimer (10 μg mL^−1^) was immobilized to ELISA wells and left overnight at 4 °C. GPVI‐Fc_2_ (20 μg mL^−1^) was incubated with increasing concentrations of collagenous substrate (0–20 μg mL^−1^) and ELISA performed as described in the Methods. The following collagenous substrates were tested: collagen type III (col III, non‐fibrous), Horm collagen (fibrous), CRP‐XL and GPP10‐XL. (A i) CRP‐XL inhibited GPVI‐Fc_2_ binding to D‐fragment, abrogating it at ≥ 0.05 μg mL^−1^ and col III and Horm, at ≥ 0.5 μg mL^−1^. GPP10‐XL, which does not bind to GPVI‐Fc_2_, did not affect the binding. (A ii) GPVI‐Fc_2_ binding to D‐dimer was similarly inhibited by col III, Horm and CRP‐XL, although slightly higher concentrations of col III and Horm were needed to obtain the same degree of inhibition. (B) Col III, Horm and CRP‐XL (10 μg mL^−1^) were immobilized to ELISA wells and left overnight at 4 °C. D‐fragment, D‐dimer, E‐fragment, fibrinogen (Fib‐3) and its respective pFibrin (0–20 μg mL^−1^) incubated with GPVI‐Fc_2_ (20 μg mL^−1^) cannot inhibit GPVI‐Fc_2_ binding to col III (B i), HORM (B ii), or CRP‐XL (B iii), suggesting that D‐domain in solution does not have sufficient affinity for GPVI‐Fc_2_. Data are presented as the mean ± SEM obtained from three separate experiments with each data point as a duplicate.

To determine if the binding site on GPVI‐dimer for D‐fragment and D‐dimer is close to that for collagen, we incubated GPVI‐Fc_2_ (20 μg mL^−1^) with 0–20 μg mL^−1^ of col III (non‐fibrous), Horm collagen (fibrous), CRP‐XL and GPP10‐XL and reacted it with immobilized D‐fragment and D‐dimer. The converse experiment, where immobilized collagenous substrates were reacted with GPVI‐Fc_2_ incubated with fibrinogen, fibrins or fibrinogen subdomains, was also performed (described in next section). Col III and Horm showed similar concentration‐dependent abilities to inhibit binding of GPVI‐Fc_2_ to D‐fragment, with complete inhibition at ≥ 0.5 μg mL^−1^ (Fig. 2A‐i). CRP‐XL was effective at lower concentrations, producing complete inhibition at ≥ 0.05 μg mL^−1^. Higher col III and Horm concentrations were needed to inhibit GPVI‐Fc_2_ binding to D‐dimer, whereas the inhibition curve of CRP‐XL was similar to that against D‐fragment (Fig. 2A‐ii). As expected, GPP10‐XL had little effect against GPVI‐Fc_2_ binding.

### Determining if GPVI‐Fc_2_ and platelet GPVI can interact with fibrinogen or its derivatives in solution

D‐fragment, E‐fragment, D‐dimer, fibrinogen and pFibrin (0–20 μg mL^−1^) could not inhibit binding of GPVI‐Fc_2_ (20 μg mL^−1^) to immobilized col III, Horm or CRP‐XL (Fig. 2B i–iii), suggesting that GPVI‐Fc_2_ cannot bind to them in solution.

Flow cytometry (Fig. 5C) shows that the washed normal platelets bind to fibrinogen and D‐dimer, at a low level, but thrombasthenic platelets that lack αIIbβ3 exhibit no binding to either. This means that activation through αIIbβ3 is required for expression of D‐dimer and fibrinogen binding; in this case, the platelets may have become slightly activated during the washing procedures.

pFibrin (> 5 μg mL^−1^) was able to induce platelet aggregation (Figure S2); mFab‐F prolonged its lag‐time but did not reduce maximum aggregation, whereas 45‐μm Eptifibatide (anti‐αIIbβ3) abrogated it. This suggests that platelet aggregation through pFibrin in suspension doesn't require GPVI‐dimer. Fibrinogen, D‐fragment and D‐dimer (5–20 μg mL^−1^) did not induce platelet aggregation (Fig. 5A‐i). However, D‐dimer concentration‐dependently inhibited Horm‐induced aggregation of washed platelets (Fig. 5A‐ii). In platelets pre‐incubated with 100 μg mL^−1^ Horm, lower D‐dimer concentrations did not affect mFab‐F inhibition of Horm‐induced aggregation, but high D‐dimer concentration relieves the inhibition, suggesting that D‐dimer may bind at a separate but nearby site, perhaps producing a conformational change that may reduce the affinity of mFab (Fig. 5A‐iii).

Therefore, fibrinogen derivatives in solution are not able to bind to platelet GPVI‐dimer or GPVI‐Fc_2_. Although D‐dimer appears to bind to GPVI‐dimer in suspension, it does not cause aggregation, and indeed inhibited it, an important physiological barrier to prevent platelets from spontaneous aggregation through GPVI‐dimer.

### Platelet adhesion to fibrinogen substrates under static conditions and effect of inhibiting antibodies

**Figure 3:**
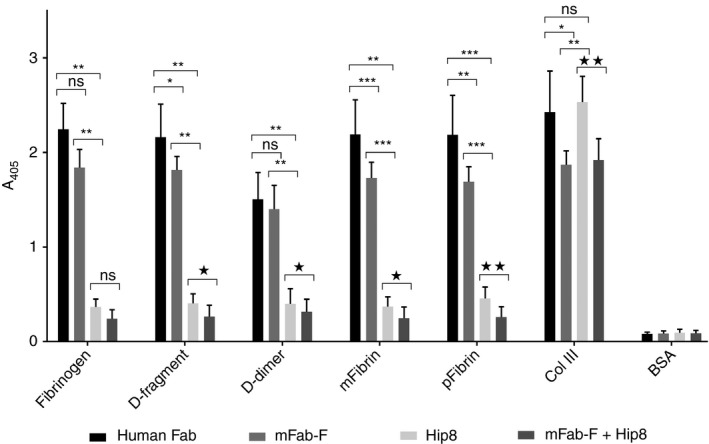
Platelet adhesion to immobilized fibrinogen substrates under static conditions and effect of antibodies against GPVI‐dimer and integrin αIIbβ3. Adhesion of washed platelets pretreated with human Fab (control, 200 μg mL^−1^), mFab‐F (200 μg mL^−1^), Hip8 (anti‐GPIIbα, inhibitory, 10 μg mL^−1^), or a combination of mFab‐F (200 μg mL^−1^) and Hip8 (10 μg mL^−1^), was determined as previously described; in this assay, the adhered platelets are lysed and their content of alkaline phosphatase measured in an assay using the chromogenic substrate *p*‐nitrophenyl phosphate, which is hydrolyzed by this enzyme to *p*‐nitrophenol, which is detectable at 405‐nm absorbance. Data shown are the mean ± SD of three separate experiments, each data point run in duplicate, using platelets from three different healthy control individuals. Data were analyzed by the paired *t*‐test, with significances shown on the figure: control (human Fab) vs. mFab‐F, Hip8 or mFab8 + Hip8 (*P < 0.05, **P < 0.01,***P < 0.001); mFab vs. Hip8 (**P < 0.01, ***P < 0.001); Hip8 vs. Hip8 + m‐Fab‐F (*P < 0.05,**P < 0.01); ns = not significant. Platelets adhere to fibrinogen, pFibrin (polymerized fibrin made from Fib‐3), mFibrin (monomeric fibrin made from Fib‐3), D‐fragment, D‐dimer and collagen type III (col III), the positive control. Platelets do not adhere to E‐fragment, so the following discussion of antibody effects only refers to the substrates supporting platelet adhesion. mFab‐F mildly but significantly decreased platelet binding to D‐fragment, m‐fibrin, p‐fibrin and collagen. Hip8 markedly decreases platelet adhesion to all fibrinogen substrates, but has no effect on platelet adhesion to collagen, which is mainly supported by integrin α2β1. Adding mFab‐F and Hip8 together further diminishes platelet adhesion to all immobilized fibrinogen substrates, suggesting that integrin αIIbβ3 plays the predominant role but GPVI‐dimer makes a significant contribution.

Under static conditions, washed platelets adhere to fibrinogen, D‐fragment, pFibrin, mFibrin and D‐dimer (Fig. 3), but not to E‐fragment (data not shown in Fig. 3). mFab‐F mildly but significantly inhibited platelet adhesion to D‐fragment, mFibrin, pFibrin and col III, whereas hFab (human Fab) had no effect. Hip8 dramatically decreased platelet adhesion on fibrinogen, D‐fragment, D‐dimer, mFibrin and pFibrin. mFab‐F  +  Hip8 further decreased platelet adhesion on D‐fragment, D‐dimer, mFibrin and pFibrin. Thus, static adhesion to fibrinogen substrates is mainly αIIbβ3‐dependent, with a small contribution by GPVI‐dimer. In contrast, static adhesion on collagen mainly depends on α2β1, with a lesser contribution by GPVI‐dimers, under the present non‐cation‐depleted conditions 4.

### Platelet spreading on immobilized fibrinogen substrates requires GPVI‐dimer

**Figure 4:**
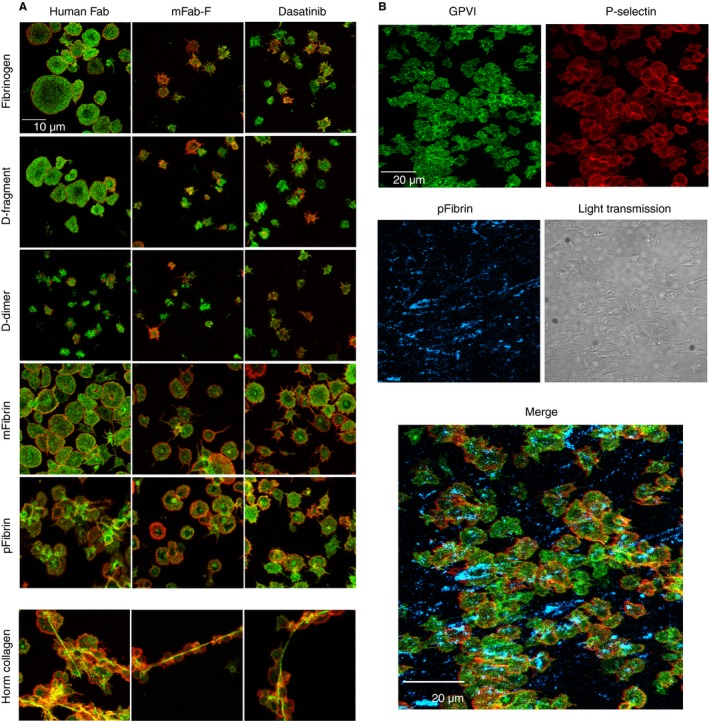
Confocal images of washed platelets adhered to fibrinogen derivatives and Horm collagen and the effect of inhibitors. Washed platelets suspended in HT buffer were pretreated with human Fab (control, 200 μg mL^−1^), inhibitory anti‐GPVI‐dimer mFab‐F (200 μg mL^−1^) or the Src inhibitor Dasatinib (50 nm) for 10 min and then were allowed to adhere to fibrinogen, fibrinogen derivative or Horm collagen immobilized on MatTek glass dishes, followed by fixation and staining with fluorescently labelled antibody (as described in the Methods). (A) Images show platelets stained with Alexa‐fluor 488–1G5 (green; pan GPVI) and Alexa‐fluor 647–anti‐P‐selectin (red) to measure platelet activation. Images were obtained with a 60x oil immersion objective on an Olympus confocal microscope. As shown in (A) (left‐most column of images), platelets spread well on fibrinogen, D‐fragment, mFibrin and Horm collagen, with evident surface expression of P‐selectin. Platelet aggregate formation was seen on pFibrin, whereas platelets spread but did not appear aggregated on mFibrin. Horm collagen supported aggregate formation, with the characteristic binding of GPVI along the Horm fibers 31. In contrast, platelets did not spread well on D‐dimer, with only some platelets showing P‐selectin expression. Treatment with mFab‐F (A, middle column of images) prevented platelet spreading on fibrinogen, D‐fragment and Horm collagen, and decreased the extent of spreading, with evident filopodia observed, on mFibrin and pFibrin, whereas there was little change in the less‐spread platelets on D‐dimer. mFab‐F prevented aggregate formation on both pFibrin and Horm collagen, consistent with inhibition of activation through GPVI. Dasatinib (A, right‐most column of images) shows effects similar to those of mFab‐F. (B) In these experiments, the immobilized pFibrin or mFibrin (data not shown) was stained with anti‐fibrin/Alexa‐fluor 547–anti‐mouse IgG (blue) after the surface was blocked with 1% bovine serum albumin; the dishes were then thoroughly washed and used for the platelet adhesion experiments, which were performed as shown in (A). pFibrin (blue) is clearly evident, whereas there was no staining of the immobilized mFibrin (data not shown). P‐selectin (red) expression and aggregate formation is evident on the surface of the adhered platelets.

Confocal images (Fig. 4A) of platelets labeled for pan‐GPVI (Alexa‐fluor 488–1G5, green) and P‐selectin (Alexa‐fluor 647–anti‐CD 62p, red) indicate that platelets spread well, with P‐selectin expression, on fibrinogen, D‐fragment, mFibrin and pFibrin, which shows clear aggregate formation. Little spreading occurred on D‐dimer. mFab‐F and Dasatinib (Src‐kinase inhibitor) cause similar inhibition of platelet spreading on fibrinogen and D‐fragment, and although platelets still spread on pFibrin, aggregate formation is obviously reduced. As expected, platelets bind through GPVI on Horm fibers, with surface P‐selectin expression, as reported before 31. Figure 4(B) shows a representative image of immobilized pFibrin fibers, pre‐stained as described in the Methods, before platelets were added, with adhered platelets showing pan‐GPVI and surface P‐selectin expression.

### Both GPVI‐dimer and integrin αIIbβ3 contribute to platelet adhesion and thrombus formation on fibrinogen derivatives under flow conditions

Human blood from a healthy donor was perfused over immobilized fibrinogen derivatives or col III (control) at 350 s^−1^ (Fig. 6) and 1000‐s^−1^ (Figure S3). Thrombus formation is apparent on all control images (left column, Fig. 6A). Addition of mFab‐F (middle column of images, Fig. 6A) decreases both platelet adhesion (SA) (Fig. 6B) and mean thrombus height (MTH) (Fig. 6C) on all tested surfaces. Eptifibatide abrogated platelet adhesion on all four fibrinogen derivatives (right column, Fig. 6A).

Inhibition of α2β1 by mAb Gi9 (10 μg mL^−1^) only mildly inhibited adhesion on collagen, but not the fibrinogen substrates. Compared with hFab (100 μg mL^−1^), GPVI‐dimer inhibition resulted in reduced SA and MTH on all fibrinogen derivatives (Fig. 6D and E). All differences were statistically significant apart from mean SA on D‐fragment and D‐dimer, which was close to reaching significance (*P = *0.07 and *P = *0.08, respectively). This confirms that GPVI‐dimer contributes to platelet adhesion and thrombus formation on immobilized fibrinogen and fibrin surfaces, although the effect of αIIbβ3 is greater.

### Assessing the role of GPVI‐dimer in platelets from a patient with Glanzmann's thrombasthenia (GT)

Because platelets contain far fewer GPVI‐dimers than integrin αIIbβ3, which may mask the subtler effects of GPVI‐dimer, we examined static adhesion and flow adhesion using blood from a GT patient, whose platelets were confirmed to have no αIIbβ3 but a normal GPVI level (Fig. 7A).

Static adhesion of GT platelets: Washed GT platelets showed essentially no adhesion on fibrinogen or D‐fragment, but retained a low level of adhesion on pFibrin and D‐dimer (Fig. 7B). mFab‐F (100 μg mL^−1^) further diminished the adhesion to pFibrin, suggesting that this low level of interaction involved GPVI‐dimer.

Confocal imaging showed that some GT platelets pre‐labelled with Alexa‐fluor 488‐conjugated 204‐11‐Fab (green) and post‐labelled with Alexa‐fluor 647‐conjugated anti‐GPIb (red) (Fig. 7C) adhered to pFibrin fibers (Fig. 7C, transmitted light image), but did not spread/aggregate like the normal platelets (post‐labelled with Alexa‐fluor 647‐conjugated anti‐αIIbβ3).

Under flow conditions, adhesion of GT platelets to D‐fragment, D‐dimer, fibrinogen and pFibrin was dramatically reduced, but platelet adhesion was still evident (Fig. 8A). Quantitation of SA (Fig. 8B) showed that GT platelets retain some ability to adhere to fibrinogen, pFibrin, D‐fragment and D‐dimer. Their SA on D‐fragment and D‐dimer was reduced by mFab‐F, suggesting GPVI‐dimer involvement in this adhesion. MTH for the GT platelets (Fig. 8C) was very low, indicating little platelet activation had occurred. This suggests that the absence of αIIbβ3 does not abolish the interaction between GPVI and fibrinogen substrates, although it appears that its absence impairs the ability of platelets to activate on fibrinogen and fibrin.

## Discussion

GPVI‐dimer, constitutively present in resting platelets 4, by virtue of its high affinity for collagen, is the functional form of this receptor that initiates signaling leading to thrombogenesis 27. Recent work has identified GPVI as a fibrin receptor. Mammadova‐Bach *et al*. 24 first showed that GPVI promoted thrombin generation through fibrinogen and fibrin‐dependent mechanisms and that Fab 9O12 (inhibitory antibody against pan GPVI) 34 reduced platelet adhesion to fibrin and platelet recruitment to formed fibrin‐rich thrombi 24. Subsequently, Alshehri *et al*. concluded that fibrin stimulates tyrosine kinase phosphorylation and platelet spreading through GPVI, independent of αIIbβ3 25, and Onselaer *et al*. recently reported that GPVI‐monomer binds to fibrin 26.

**Figure 5:**
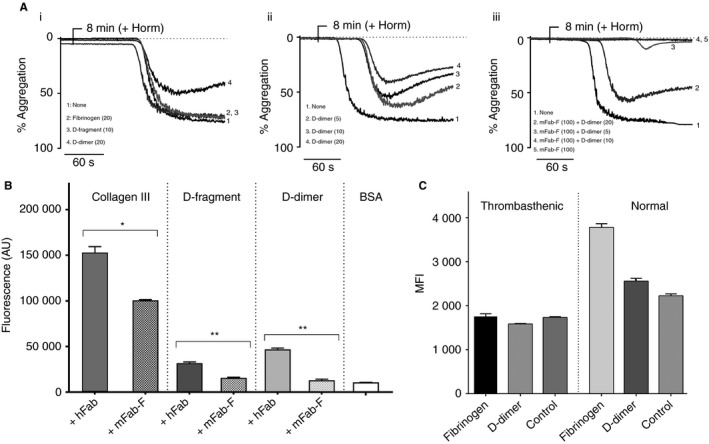
Binding of GPVI‐dimer to D‐fragment, D‐dimer, fibrinogen and fibrin in solution and effect of mFab‐F on static adhesion to D‐domain. (A) Aggregometry was performed with washed platelets (2.5 × 10^8^) either untreated or incubated for 5 min with mFab‐F (100 μg mL^−1^)). At 3 min, D‐fragment, D‐dimer or fibrinogen (parentheses indicate concentrations in μg mL^−1^) was added to the platelets (A i–iii). D‐fragment, D‐dimer or fibrinogen do not cause aggregation up to the maximum concentration (20 μg mL^−1^). If no aggregation occurred by 8 min, Horm was added to induce platelet aggregation, verifying that the platelets were active. D‐dimer inhibits Horm‐induced aggregation (A i) in a concentration‐dependent manner (A ii). (A iii) Washed platelets were pre‐incubated with 100 μg mL^−1^ of mFab‐F for 5 min. D‐dimer (5–20 μg mL^−1^) was added at 3 min and Horm (3 μg mL^−1^) at 8 min. Horm‐induced aggregation is inhibited when mFab‐F is present, although at higher concentrations of D‐dimer (20 μg mL^−1^) some aggregation occurs. The images are representative of results from three experiments. (B) ELISA plates were coated with col III, D‐fragment or D‐dimer (25 μg mL^−1^) and left overnight at 4 °C. GPVI‐Fc_2_ (25 μg mL^−1^) was incubated with either 100 μg mL^−1^ human Fab (hFab), as a control, or 100 μg mL^−1^ mFab‐F for 1 h at room temperature and reacted with the coated substrates. Bound GPVI‐Fc_2_ was detected as detailed in the Methods. Results, presented as the mean ± SEM, confirm that mFab‐F significantly reduces GPVI‐Fc_2_ binding to D‐fragment, D‐dimer *(P < *0.01) and collagen (*P < *0.05). (C) Flow cytometry assessment of fibrinogen and D‐dimer binding in normal and GT platelets, with isotype control (IgG1). Washed platelets from the GT patient and a normal donor were incubated with 20 μg mL^−1^ of fibrinogen or D‐dimer for 30 min; samples were diluted 10‐fold with CBS, pelleted by centrifugation and resuspended in HT; then each sample was incubated with anti‐human fibrinogen for 10 min, followed by addition of an excess of Alexa fluor‐488–anti‐mouse Fab. Samples were measured in an Accuri C6 flow cytometer. The normal platelets show low levels of fibrinogen and D‐dimer binding, but the GT platelets show neither, suggesting that integrin αIIbβ3 is mainly responsible for binding to fibrinogen and D‐dimer.

The present study presents the novel finding that GPVI‐dimer, not the monomer, binds to immobilized fibrinogen and fibrin through their D‐domains. GPVI‐dimer binds to the D‐domain with high affinity, exhibiting *K*_d_ values similar to that for collagen (Fig. 1A, E). We found that GPVI‐monomer bound to none of the fibrinogen derivatives (Fig. 1B), confirming a specific interaction between GPVI‐dimer and the D‐domain. Neither recombinant nor platelet GPVI‐dimer binds E‐fragment. Interestingly, the ability of collagenous substrates to inhibit GPVI‐dimer binding to D‐fragment and D‐dimer (Fig 2A), as well as the ability of soluble D‐dimer to inhibit collagen‐induced platelet aggregation (Fig 5A‐ii), suggests that the collagen and D‐domain binding sites on GPVI‐dimer are proximate.

The collagen‐binding properties of the GPVI‐ectodomain used by the Watson group (Alsheri *et al*. 25) initially have not been characterized, so we are unable to compare it with our GPVI_ex_. Subsequently, they (Onselaer *et al*. 26) used recombinant GPVI proteins and concluded that GPVI‐monomer selectively binds to fibrin. We found the opposite to be true: only GPVI‐dimer binds to the D‐domain common to fibrinogen and its derivatives, consistent with the specific binding of the recombinant GPVI‐Fc to fibrin reported by Mammadova‐Bach *et al*. 24. These discrepancies would come from the difference between our GPVI recombinant proteins and those used by the Watson group: their recombinant proteins do not include the highly glycosylated, sialic‐acid‐rich stem portion of GPVI and the lack of such strong anionic charges would influence the binding of their recombinant GPVI to fibrin. To obviate the potential problem caused by the lack of carbohydrate in their constructs, they showed that Revacept, a dimeric GPVI‐Fc‐fusion protein, also did not bind fibrin. However, Revacept has a different structure from platelet GPVI‐dimer and our recombinant dimer because a flexible linker sequence has been inserted between the GPVI‐extracellular domain and the Fc portion 35. This may make its conformation different from that of physiological GPVI‐dimer and thereby change its ability to bind to fibrin. It must be highlighted that our antibodies 204‐11 Fab and mFab‐F, designed against our GPVI‐Fc_2_, are able to recognize both the constitutive dimers present on resting platelets and those on activated platelets and mFab‐F inhibits Horm‐induced aggregation, supporting that our GPVI‐Fc_2_ is similar to physiological platelet GPVI‐dimers.

A key finding from our work is that the immobilized nature of the fibrinogen substrates is important for platelet activation through GPVI‐dimer. Fibrinogen subdomains in solution with GPVI‐dimer cannot inhibit its binding to collagen (Fig. 2B) and GT platelets lacking αIIbβ3 fail to bind fibrinogen and D‐dimer in solution (Fig. 5C). Aggregometry confirms that D‐fragment, D‐dimer and fibrinogen in solution do not cause platelet aggregation (Fig. 5A‐i), and pFibrin suspension induces platelet aggregation independent of GPVI‐dimer, presumably via αIIbβ3, as mFab‐F increased the lag‐time, but did not affect the level of maximum aggregation (Figure S2). Together, these results suggest that GPVI‐dimer cannot bind to fibrinogen or its subdomains to cause activation unless they are immobilized. This is crucial as platelet aggregation through binding to soluble fibrinogen does not occur in the circulation under normal conditions, unless initial platelet activation has occurred, converting resting αIIbβ3 to a high‐affinity state 36.

Although the fibrinogen and its subdomains in solution cannot activate platelets, D‐dimer, which is found in plasma at high concentrations under pathological conditions as a fibrinolytic product, is inhibitory towards platelet activation through GPVI. Lee *et al*. 37 reported the ability of soluble, crosslinked fibrin, which contains the D‐dimer moiety, to inhibit platelet activation through GPVI‐signaling and we and Onselaer *et al*. 26 both found that D‐dimer inhibits collagen‐induced platelet aggregation.

GPVI‐dimer contributes significantly to static platelet adhesion, independently of αIIbβ3, on immobilized D‐fragment, pFibrin and mFibrin (Fig. 3). Furthermore, mFab‐F inhibition of GPVI‐Fc_2_ leads to a significant reduction in its binding to D‐fragment, D‐dimer and col III in ELISA (Fig. 5B). GPVI‐dimer not only contributes to adhesion but also to spreading on fibrinogen, D‐fragment, mFibrin, and pFibrin, and aggregate formation on pFibrin (Fig. 4).

**Figure 6:**
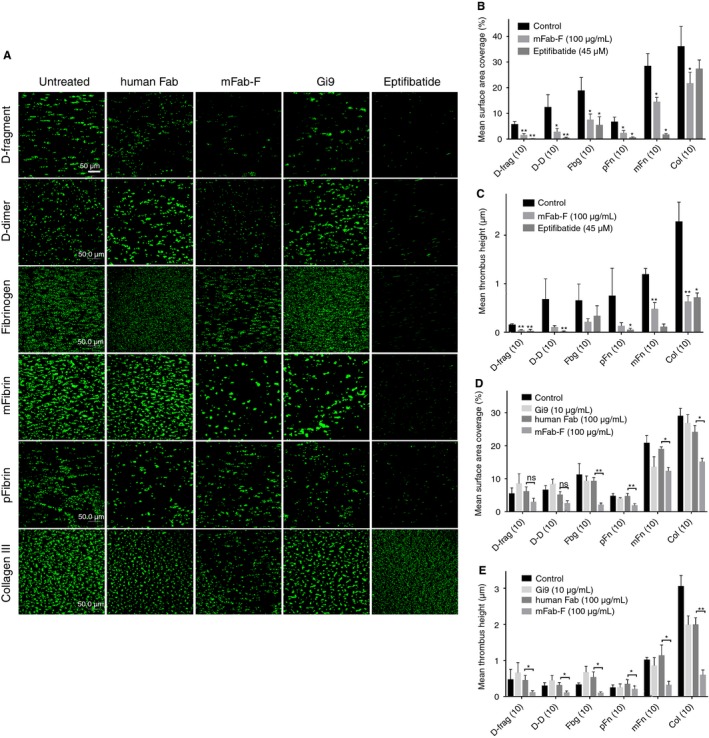
Adhesion of platelets in whole blood to fibrinogen substrates and collagen under flow conditions. The following abbreviations were used in the graphs (B–E): D‐fragment (D‐frag), D‐dimer (D‐D), fibrinogen (Fbg), pFibrin (pFn), mFibrin (mFn) and collagen type III (col). Glass coverslips were coated with fibrinogen (Fbg‐3), pFibrin and mFibrin (produced from Fbg‐3), D‐fragment, D‐dimer and collagen type III at 10 μg mL^−1^ in PBS and left overnight at 4 °C. Blood was anticoagulated with 36 μL D‐phenylalanyl‐prolyl‐arginyl chloromethyl ketone (PPACK) and topped up at 1 : 1000 ratio every hour. Platelets in whole blood were fluorescently labelled with 1:4500 DiOC6 (3,3′‐dihexyloxacarbocyanine iodide). After 1‐min perfusion of flow buffer (136 mm NaCl, 2.7 mm KCl, 5 mm *N*‐2‐hydroxyethylpiperazine‐*N*’‐2‐ethanesulfonic acid, 10 mm glucose, 2 mm MgCl_2_, 2 mm CaCl_2_, pH 7.4), whole blood was perfused over the coverslips at a shear rate of 350 s^−1^. Z‐stack images were used to calculate surface coverage (SA) of adhered platelets and mean thrombus height (MTH) in panels B–E. (A) Fluorescently labelled whole blood was left untreated (control) or treated with one of the following: human Fab (hFab, 100 μg mL^−1^), mFab‐F (GPVI‐dimer specific, inhibitory; 100 μg mL^−1^), Eptifibatide (αIIbβ3 inhibitor, 45 μm; Tocris Bioscience, UK), Gi9 (anti‐α2β1, inhibitory; GeneTex, USA). The images in Panel A are representative of six flow experiments performed with blood from different donors. Statistical significance between control and antibody inhibition (B + C) or human Fab and mFab‐F inhibition (D + E) was calculated using paired *t*‐tests (*P < 0.05, **P < 0.01 ***P < 0.001). (B + C) Inhibition of αIIbβ3 abrogated SA and MTH to all surfaces. mFab‐F reduced platelet adhesion (SA) to all five fibrinogen derivatives (D‐fragment, **P < 0.01; all others, *P < 0.05) and col III (*P < 0.05). mFab‐F also significantly suppressed MTH on D‐fragment, mFibrin and col type III (**P < 0.01) and reduced it on D‐dimer, fibrinogen and pFibrin compared with the control. This suggests that GPVI‐dimer is able to facilitate platelet adhesion and thrombus growth on fibrinogen derivatives, through the D‐domain. (C + D) The inhibition of GPVI‐dimer with mFab‐F compared with the effect of human Fab and anti‐α2β1 Gi9 under the same conditions. α2β1 had little to no effect on adhesion to fibrinogen derivatives, and as expected caused some reduction of aggregate formation (MTH) on collagen. mFab‐F significantly reduced SA and MTH compared with human Fab on all tested substrates, except SA on D‐fragment and D‐dimer, which nevertheless tended towards significance (*P *= 0.07 and *P* = 0.08, respectively).

Platelet adhesion to fibrinogen substrates under flow is more complex than measuring direct binding using ELISA, because it involves a dominant role for αIIbβ3 at lower (350 s^−1^) and higher (1000 s^−1^) shear, as demonstrated by the abrogation of thrombus formation in the presence of Eptifibatide (Fig. 6, Figure S3). GPVI‐dimer, however, appears to play its most significant role at low shear, because mFab‐F significantly decreases SA on all tested substrates and MTH is significantly reduced on D‐fragment, mFibrin and collagen (Fig. 6, Table S1). At 1000 s^−1^, GPVI inhibition appears to produce more variable results, which suggests that GPVI‐dimer is less able to recognize immobilized D‐domain at higher shear. This indicates that αIIbβ3 does play a significant role in platelet interactions with fibrinogen and fibrin in both static (Fig. 3) and shear (Fig. 6) conditions, whereas GPVI‐dimer has a secondary but requisite role for inducing platelet adhesion and thrombus formation.

This could be through convergent tyrosine kinase signaling downstream from GPVI and αIIbβ3. For example, the crosslinking of GPVI induces Src‐dependent tyrosine phosphorylation of FcRγ 2 and, similarly, signaling mediated by αIIbβ3 also includes tyrosine kinases such as Syk and Src 38, 39, with specific blockade reducing the tyrosine phosphorylation of signaling proteins downstream from Syk in both the GPVI and αIIbβ3 signaling pathways 40. Dasatinib and mFab‐F each exert a similar inhibitory effect on platelet adhesion, indicating platelet‐adhesion‐induced signaling through GPVI‐dimer is an important step in platelet spreading and activation on fibrinogen and fibrin (Fig. 4). This is consistent with Onselaer *et al*., who show that fibrin is unable to activate GPVI‐deficient human platelets, which is further evidence of shared signaling pathways between GPVI and αIIbβ3 26.

**Figure 7:**
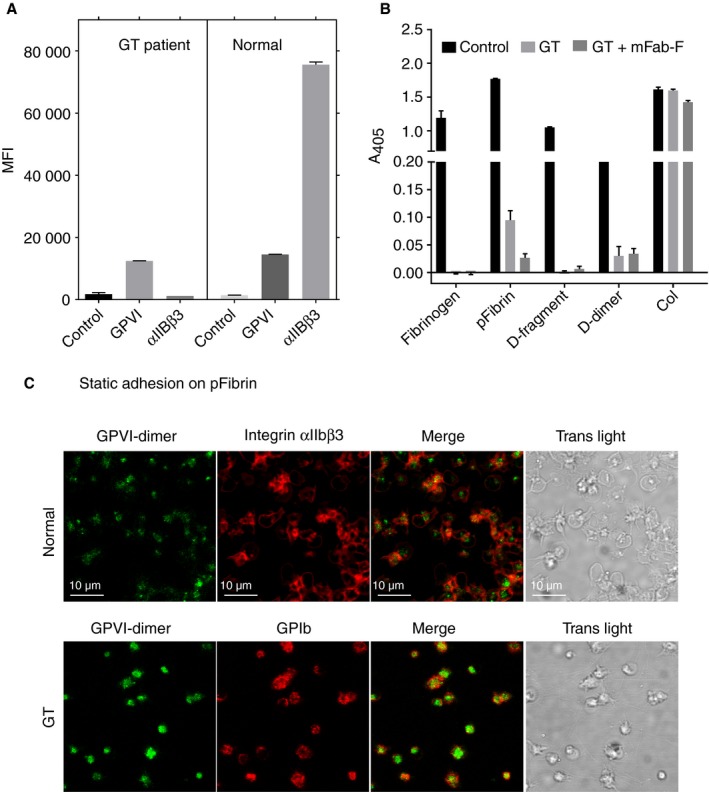
Static adhesion of platelets from an individual with Glanzmann thrombasthenia (GT) on immobilized fibrinogen substrates. (A) Flow cytometry analysis verified that the GT platelets contain no integrin αIIbβ3, but had normal levels of GPVI. (B) In these experiments, the ELISA wells were coated by adding a 100‐μg mL^−1^ solution of each substrate. The GT platelets did not adhere to fibrinogen or D‐fragment, but retained the ability to adhere to pFibrin and D‐dimer, although this was markedly reduced. mFab‐F markedly reduced the adhesion on pFibrin, suggesting that GPVI‐dimer may be involved in this interaction. (C) Confocal images of normal platelet and GT platelets adhered to immobilized pFibrin. Both normal and GT platelets were prelabelled with Alexa fluor 488‐conjugated 204‐11 Fab and allowed to adhere on pFibrin‐coated MatTek dishes, for 30 min at 37 °C. The platelets were then fixed and stained with Alexa fluor 647‐conjugated antibody specific for integrin αIIbβ3 (normal platelets) or GPIb (GT platelets), as described in the text, before imaging. The GT platelets retain the ability to adhere to pFibrin, although they do not spread; close inspection of the transmitted light images shows that the GT platelets adhere on or close to the fibers of pFibrin.

**Figure 8:**
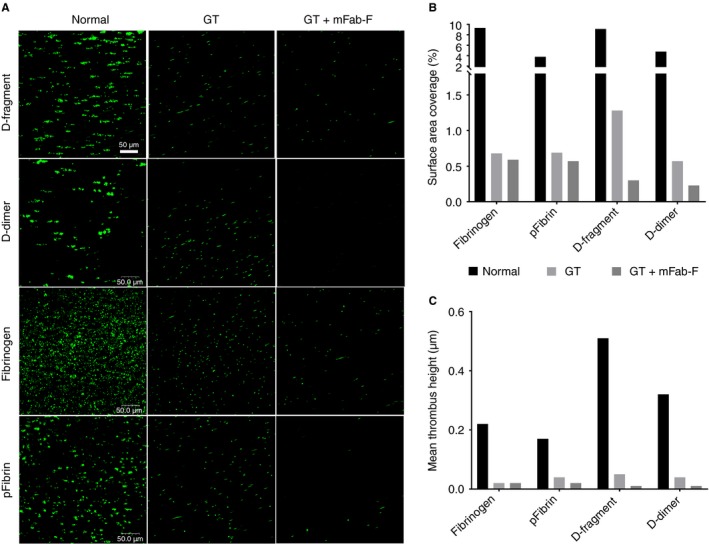
Analysis of Glanzmann thrombasthenia (GT) platelets under flow conditions. Blood was obtained from an individual with GT, whose platelets lack integrin αIIbβ3. (A) Labelled whole blood was then perfused over surfaces of D‐fragment, D‐dimer, fibrinogen and pFibrin (slides were coated with 100 μg mL^−1^ of substrate in PBS, overnight at 4 °C). Whole blood was obtained from a healthy volunteer for control measurements. Thrombasthenic blood was perfused with or without the addition of mFab‐F (100 μg mL^−1^) at 350 s^−1^. GT platelets retain some ability to adhere on all four surfaces, albeit at dramatically reduced levels compared with normal platelets and there is no aggregate formation. mFab‐F further reduced the level of adherent GT platelets. Z‐stack images were used to calculate SA (B) and MTH (C). For the GT platelets, inhibition of GPVI‐dimers by mFab‐F causes a further reduction of platelet adherence and activation on D‐fragment and D‐dimer.

Under static conditions, αIIbβ3‐deficient GT platelets can adhere to pFibrin, but are not activated (Fig. 7). Under flow, the GT platelets show drastically reduced adhesion to D‐fragment, D‐dimer, fibrinogen and pFibrin, but residual adherent but non‐spread platelets remain (Fig. 8). mFab‐F reduces GT platelet adhesion on all substrates tested, suggesting that GT platelets can bind independently through GPVI‐dimer, even without functional αIIbβ3, but these adhered GT platelets appear neither spread nor activated, suggesting that signaling may not occur without the assistance of αIIbβ3. This means that platelet surface GPVI‐dimers may support a weak interaction with immobilized fibrinogen or fibrin, but it is not sufficient to initiate platelet activation. It is more likely that αIIbβ3 and GPVI‐dimer have collaborative roles in platelet activation on fibrinogen and fibrin, especially as platelets treated with both mFab‐F and Hip8 show a further decrease in adhesion compared with Hip8 alone (Fig. 3).

GPVI antagonism could provide highly specific pharmacological drugs that prevent thrombus formation in acute coronary syndrome 41 and ischemic stroke 42. This study is the first to describe an interaction between GPVI‐dimer and fibrinogen and fibrin. GPVI‐dimer binds to the D‐domain and is involved in signaling, leading to platelet spreading and activation on fibrinogen substrates, in conjunction with αIIbβ3 and independent of collagen exposure. GPVI‐dimer clearly plays a key part in *both* initiation and propagation of thrombosis at sites of atherosclerotic plaque rupture through collagen and fibrin. However, our findings also intimate a role for GPVI‐dimer in conditions where blood clots form without collagen exposure, including atrial fibrillation and deep vein thrombosis, making it an even more tantalizing pharmacological target.

## Addendum

I. Induruwa designed and performed experiments, including flow adhesion studies, prepared the figures and wrote the manuscript. M. Moroi performed the flow cytometry studies, interpreted data, wrote the manuscript and critically read the manuscript. A. Bonna and J.‐D. Malcor synthesized CRP‐XL and GPRP. J.‐M. Howes performed flow adhesion studies. E. A. Warburton read the manuscript and mentored I. Induruwa. R. W. Farndale discussed the data with us all and critically read the manuscript. S. M. Jung, the corresponding and senior author, designed and performed experiments, interpreted data, prepared the figures and wrote the manuscript.

## Disclosure of Conflict of Interests

The authors state that they have no conflict of interest.

## Supporting information


**Fig. S1.** The table describes the commercial fibrinogens and fibrinogen derivatives that were examined in our preliminary experiments.

**Fig. S2.** pFibrin causes platelet aggregation through a mechanism independent of GPVI‐dimer.

**Fig. S3.** Adhesion of platelets in whole blood under flow conditions using a shear rate of 1000 s^−1^.

**Table S1.** Comparing thrombus formation at low (350 s^−1^) and high shear (1000 s^−1^).
