# Supplementary figures and images for: Platelet collagen receptor Glycoprotein VI‐dimer recognizes fibrinogen and fibrin through their D‐domains, contributing to platelet adhesion and activation during thrombus formation
Source: J Thromb Haemost. 2018 Jan 15;16(2):389–404. doi: 10.1111/jth.13919 (PMC5838801; doi:10.1111/jth.13919)

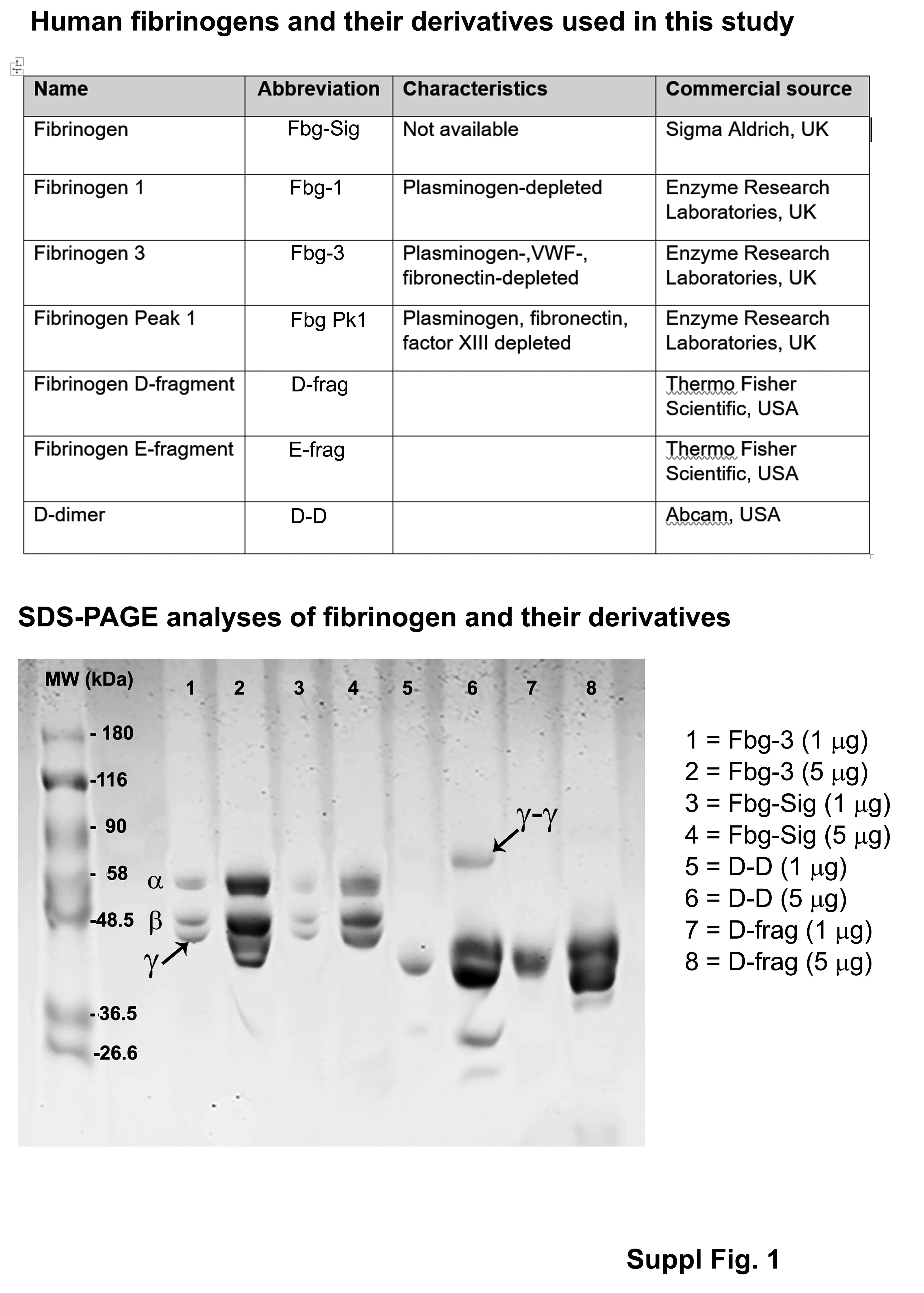

Supplement: Supplementary file 1 — Fig. S1. The table describes the commercial fibrinogens and fibrinogen derivatives that were examined in our preliminary experiments. [file JTH-16-389-s001.tif]

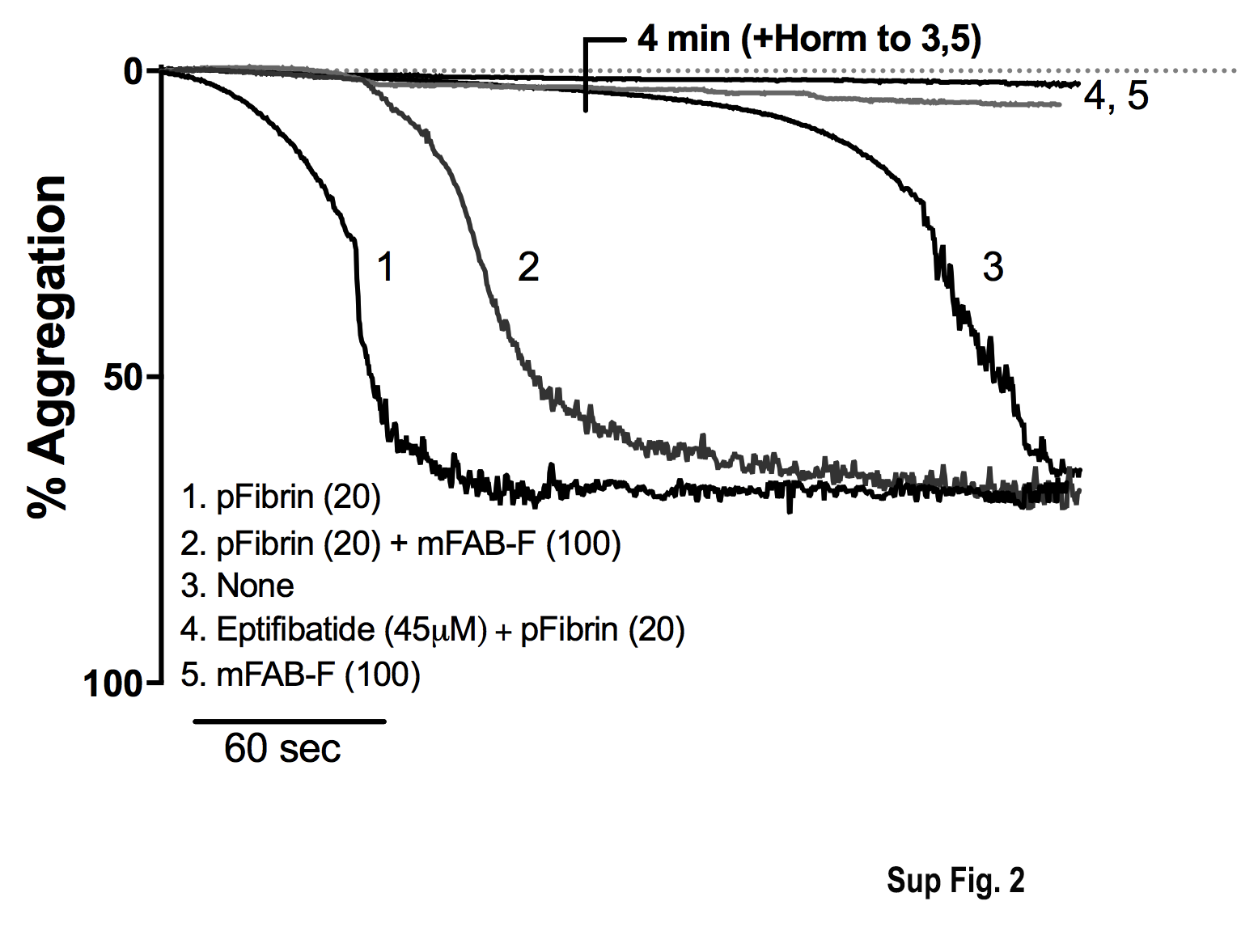

Supplement: Supplementary file 2 — Fig. S2. pFibrin causes platelet aggregation through a mechanism independent of GPVI‐dimer. [file JTH-16-389-s002.tif]

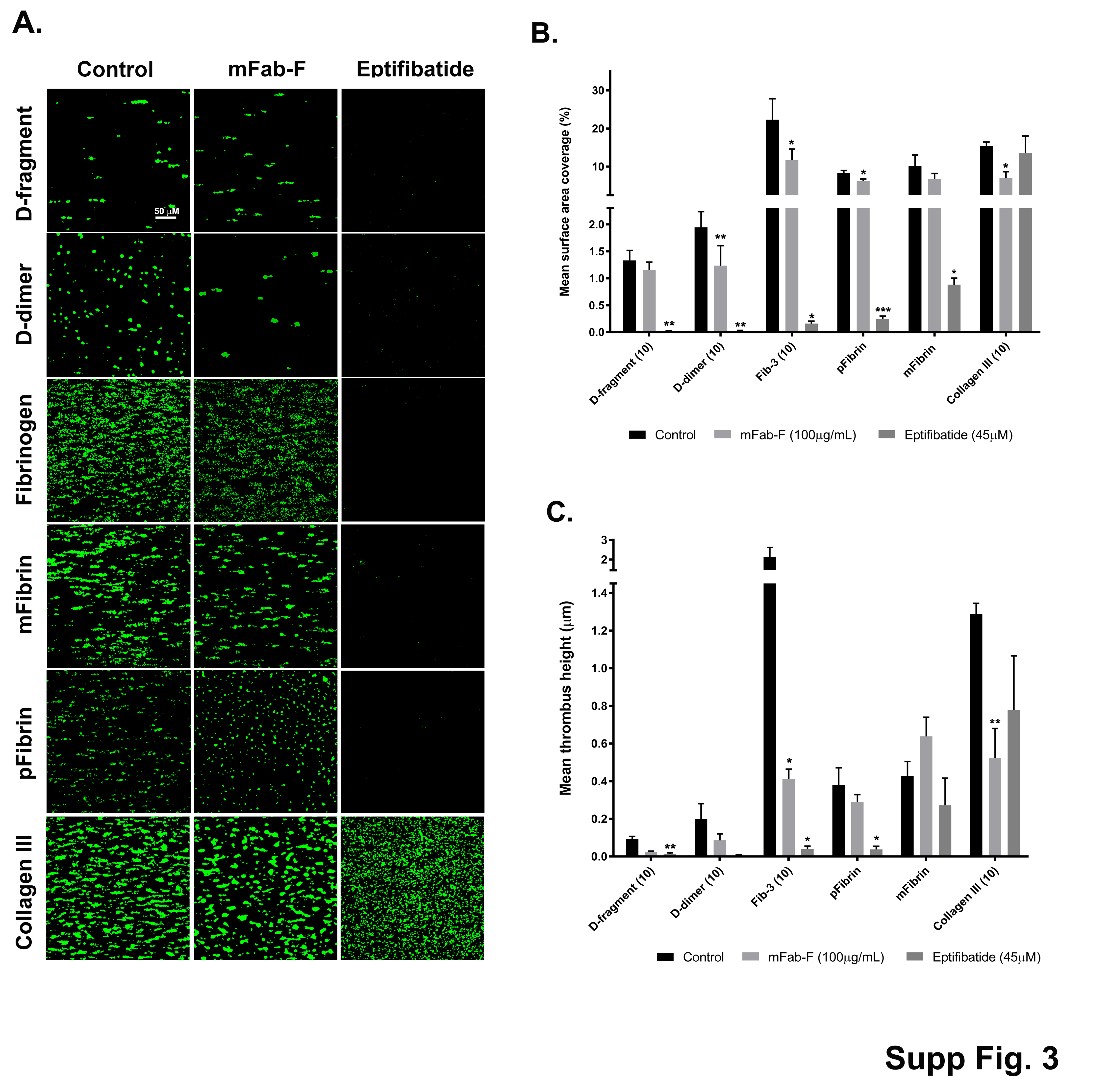

Supplement: Supplementary file 3 — Fig. S3. Adhesion of platelets in whole blood under flow conditions using a shear rate of 1000 s−1. [file JTH-16-389-s003.tif]
