# Supplementary material for: Platelet collagen receptor Glycoprotein VI‐dimer recognizes fibrinogen and fibrin through their D‐domains, contributing to platelet adhesion and activation during thrombus formation
Source: J Thromb Haemost. 2018 Jan 15;16(2):389–404. doi: 10.1111/jth.13919 (PMC5838801; doi:10.1111/jth.13919)
Supplement: Supplementary file 4 — Table S1. Comparing thrombus formation at low (350 s−1) and high shear (1000 s−1). [file JTH-16-389-s004.docx]

**Supplementary Table 1**

| % Reduction using mFAB-F compared to control *(statistical significance)* | | | | |
| --- | --- | --- | --- | --- |
|  | SA 350 s^-1^ | SA 1000 s^-1^ | MTH 350 s^-1^ | MTH 1000 s^-1^ |
| D-fragment | 71.4 *(P=0.004)* | 14.7 (ns) | 74.4 *(P=0.002)* | 74.4 *(P=0.08)* |
| D-dimer | 76.5 *(P=0.04)* | 30.7 *(P=0.005)* | 85.2 (ns) | 60 *(P=0.09)* |
| Fibrinogen | 59.*8 (P=0.03)* | 48.6 *(P=0.02)* | 68.7 (ns) | 86.6 *(P=0.02)* |
| pFibrin | 54.5 *(P=0.03)* | 32.5 *(P=0.04)* | 81.9 (ns) | 24.4 (ns) |
| mFibrin | 43.4 *(P=0.04)* | 16.5 (ns) | 50.7 *(P=0.003)* | Increase (ns) |
| Collagen III | 37.2 *(P=0.01)* | 34.2 *(P=0.01)* | 73.6 *(P=0.009)* | 44.4 *(P=0.003)* |
